# Policy Series: Update on Polling and Policy Efforts on Loneliness, Telehealth, Caregiving, and Advance Care Planning

**DOI:** 10.1093/geroni/igab046.670

**Published:** 2021-12-17

**Authors:** Erica Solway, Brian Lindberg

## Abstract

Older adults and their caregivers experienced dramatic changes in many aspects of their lives during the COVID-19 pandemic which resulted in important shifts in organizational and federal priorities and policies. To explore older adults’ changing experiences and perspectives amidst the pandemic, the University of Michigan National Poll on Healthy Aging (NPHA), a recurring, nationally representative household survey, polled over 2,000 adults age 50-80 at multiple timepoints through January 2021 about their feelings of loneliness and use of telehealth. In June 2020, the NPHA also surveyed adults age 50-80 about advance care planning before and during the COVID-19 pandemic and asked family caregivers about their care challenges in the three months since the pandemic. This session will start with a presentation of results from these polls, first exploring change over time in loneliness and telehealth use and then focusing on experiences related to advance care planning and caregiving challenges. Next, presenters from diverse national coalitions and organizations, including the Coalition to End Social Isolation and Loneliness, the National Academy for State Health Policy, the National Alliance for Caregiving, and the Coalition to Transform Advanced Care will describe their organizations’ efforts, including their work with research and advocacy partners, state and federal agencies, and the Biden administration to facilitate dialogue and advance activities and policies related to these timely topics.

